# A modern method of treatment: The role of silver dressings in promoting healing and preventing pathological scarring in patients with burn wounds

**Published:** 2016

**Authors:** A Munteanu, IP Florescu, C Nitescu

**Affiliations:** * Department of Plastic, Reconstructive Surgery and Burns, Clinical Emergency Hospital for Plastic, Reconstructive Surgery and Burns, Bucharest, Romania; ** Department of Plastic Surgery and Reconstructive Microsurgery, ”Bagdasar-Arseni” Clinical Emergency Hospital, Bucharest, Romania

**Keywords:** silver dressings, burns, preventing pathological scars

## Abstract

Burn wounds are a global public health problem, which affects all countries, no matter the development stage and occurs in all age groups, from toddlers to elderly. In spite of burns being the cause of numerous household and work accidents, there are still no clear stated unanimous rules for their treatment. Every day new products appear on the market, each of them trying to prove more effective. Since ancient times, silver has been known for its antimicrobial properties, so it has been used for a long time in the treatment of burns and other types of wounds. One of the relatively modern methods of treatment is applying silver sheets on the scald lesions.

In this paper, which was part of a larger study (research for a PhD thesis), concerning prevention and treatment of the post-burn pathological scars, the cases of some patients with burns, who were treated by using the above mentioned method were presented and analyzed. The results obtained by applying silver sheets were then commented and interpreted, pointing out the advantages and disadvantages compared to silver sulfadiazine creams and ointments, which have already been used at a large scale.

The prevention and treatment of post-burn pathological (hypertrophic and keloid) scars is a field in which still little is known and in which there are also no clearly set therapy plans. We hope that through this research and the following ones we will manage to establish some major guidelines concerning the prevention of pathological scars, which are not only disabling, but also a major aesthetic issue for any patient, in order to obtain better outcomes.

## Introduction

According to WHO (World Health Organization) burns cause “an estimated of 265000 deaths every year”. WHO also states the following: “the non-fatal burn injuries are a leading cause of morbidity”, while most of them “occur mainly in the home and workplace. Burns are preventable [**1**]”. Starting from these facts and adding the fact that burns are lesions that affect millions of people yearly worldwide, we realized that even if these accidents were preventable, they would still happen a lot. Therefore, plastic surgeons from everywhere have to treat them. It is interesting that nowadays there has been a shift in our goals: we are not only trying to rescue the patient’s life, as it used to happen in the past, but one of our main purposes is to minimize the individual’s morbidity and to obtain an aesthetic result, thus facilitating the patient’s social and professional reintegration into every-day life.

Burn treatment is very complex: it involves not only applying different topical creams/ ointments or dressings, but also the right decision-making, and this not only once, but also through the entire healing process. Every day a plastic surgeon that treats burn wounds must answer to the following questions and make the right choice, in order for the patient not to get infected, to heal quickly, and with minimal scarring: is this burn wound going to heal without any surgery? If an operation is needed, when is the right time to perform it? Are the scars of this patient going to be “pretty” ones, or will he need additional surgical procedures? Is the patient going to have any benefits after surgery, or he will only get extra scars (because surgical procedures can result in more conspicuous scars and because he is going to have scars in the donor area, too)?

Luckily, in the attempt of healing a patient the plastic surgeon nowadays has a very wide range and varied choice of modern, new dressing types that he can use. One of the most efficient categories is the one based on silver ions. These dressings have proved to be very effective not only as antimicrobial topical agents, but also in promoting healing as well.

## Case reports

In the following, the treatment plan used and the outcome in some patients with severe burns were presented. These patients were admitted in our unit – The Emergency Hospital for Plastic, Reconstructive Surgery and Burns, Bucharest – and were treated for several days until their discharge. Afterwards they came for periodical check-ups. They all had in common the fact that they displayed severe scald burns, on more than 25% TBSA (Total Body Surface Area) and that silver sheets were used in their treatment.

The purpose of this paper was to show our experience with silver -foil like- dressings and to point out the advantages and disadvantages encountered when applying this modern method of treatment. A dynamic evolution of the burn wounds in the healing process and of the resulting scars was also shown.

The principle common to all silver based dressings is that they are multilayered mesh-like sheets that are applied on the burn wound and release silver ions acting like a barrier against contamination and at the same time absorbing the exudate (which is sometimes transformed into a gel-like matter, inside the layers of the dressing), or letting it out. Thus, they keep the burn wound clean and the level of moist adequate for healing, stimulating the repair of the tissues. 

**Case 1**

**Fig. 1:**
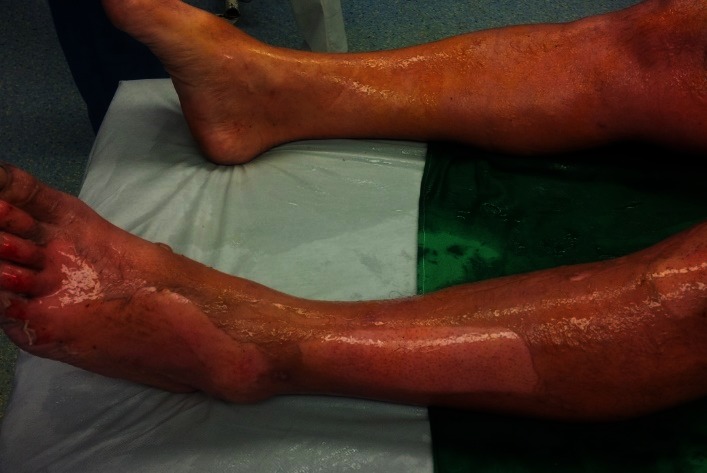
2nd A and B degree burn lesions on lower left limb, a couple of hours after the accident, pink moist, very painful

**Fig. 2:**
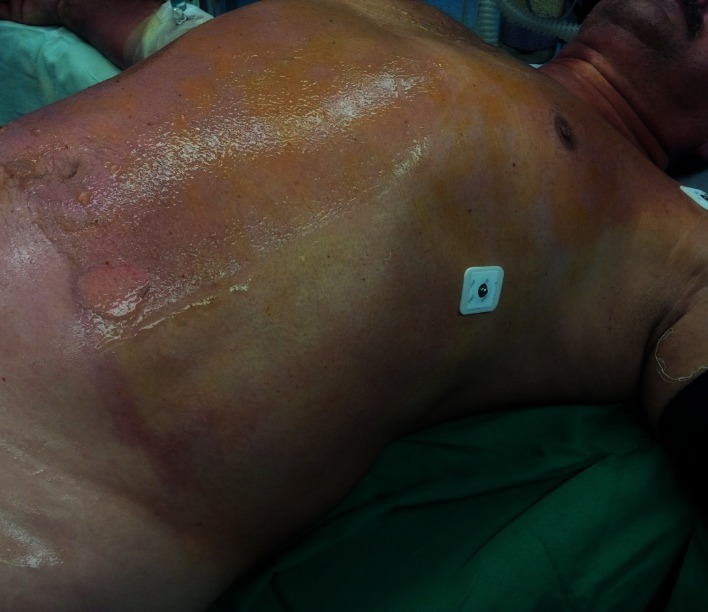
1st, 2nd A and B degree burns on anterior trunk, a couple of hours after the accident, pink moist, very painful

Patient M.F., male, aged 56 was admitted with burns of I, II and small areas of III degree on ~75% TBSA. He had the following co-morbidities: a minor liver dysfunction, due to regular alcohol consumption; untreated hypertension; type II diabetes, under treatment with Metformin; a urethral stricture, which had been surgically treated. He was a very active man, due to his job – a firefighter-. The accident happened while he was preparing some kind of alcoholic beverage at home in a special boiler, so he was splashed by the hot boiling fluid, which resulted in the injuries, which were described below. He was brought to our unit after a couple of hours from the accident displaying circular burns on both lower extremities (from the lower half of the thighs to the foot), and insular ones on his trunk, his right arm and both his hands (**Fig. 1**,**2**). No escharotomies were performed, as they were not necessary, but the surgical debridement of the lesions was immediately performed.

**Fig. 3:**
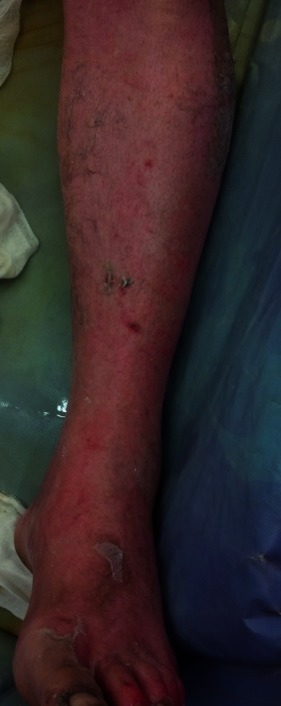
Lower left limb with healing burns, after a couple of days from entering our unit

**Fig. 4:**
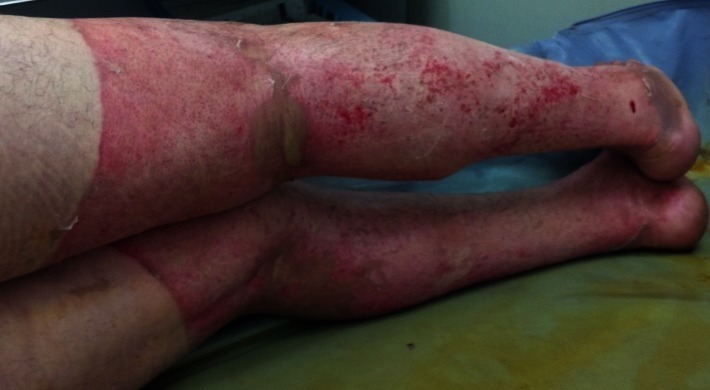
Lower limbs with healing burns, after a couple of days from admission. We observed that the burns were dry and there were small epithelization isles

After removing the blisters and the dirt, there were areas of red-pink and white dermis that was moist in some regions and dry in others. The image was that of a “chess-board”, due to the color alternation, suggesting the alternation between different degrees of burn lesions. The patient was kept, treated, and monitored in the intensive care unit for ~ 2 weeks. At some point he needed intubation and ventilator support, but his local evolution was very good. In the first couple of days, while the burn wounds were very exudative, an ointment based on zinc oxide (that was prepared in our unit) was applied (**Fig. 3**,**4**).

After the first week, when his general state got better, silver sheets (Acticoat) were applied on his trunk and on his right upper extremity, where the lesions were deeper and seemed to heal slower than in the other above-mentioned areas. They were kept in place for 3-5 days, depending on the progress of the area. In the meantime, the patient got better and better and was transferred to the regular burn department.

**Fig. 5:**
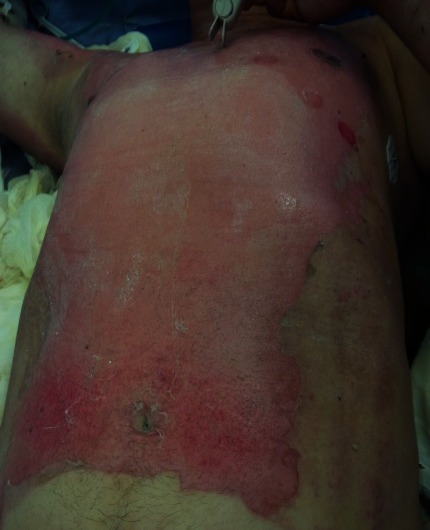
Burn wounds on the anterior trunk healed completely - this picture was taken when removing Acticoat (silver sheet dressing) – 4 days after having applied it

**Fig. 6:**
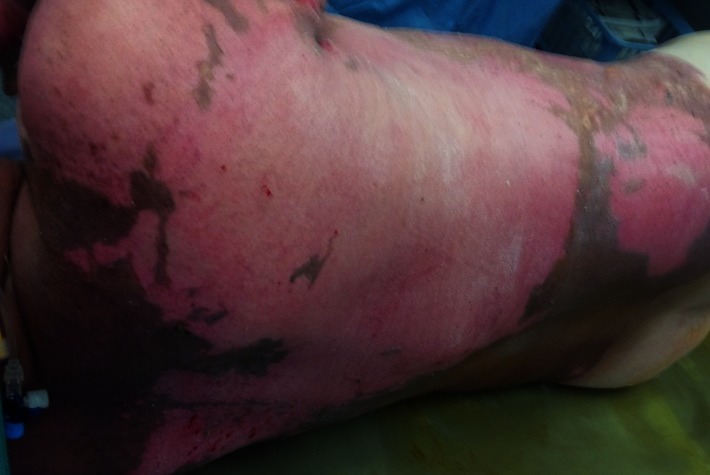
Posterior trunk healed - after removing Acticoat dressings (~5 days after having applied them)

At that point, the burns on the patient’s lower extremities were already healed, and the ones on his trunk and upper extremity were healed ~60% but he suffered from a mental blockage and he refused to get up and walk or do anything else by himself, he needed help even during the meals (**Fig. 5**,**6**). 

**Fig. 7-9:**
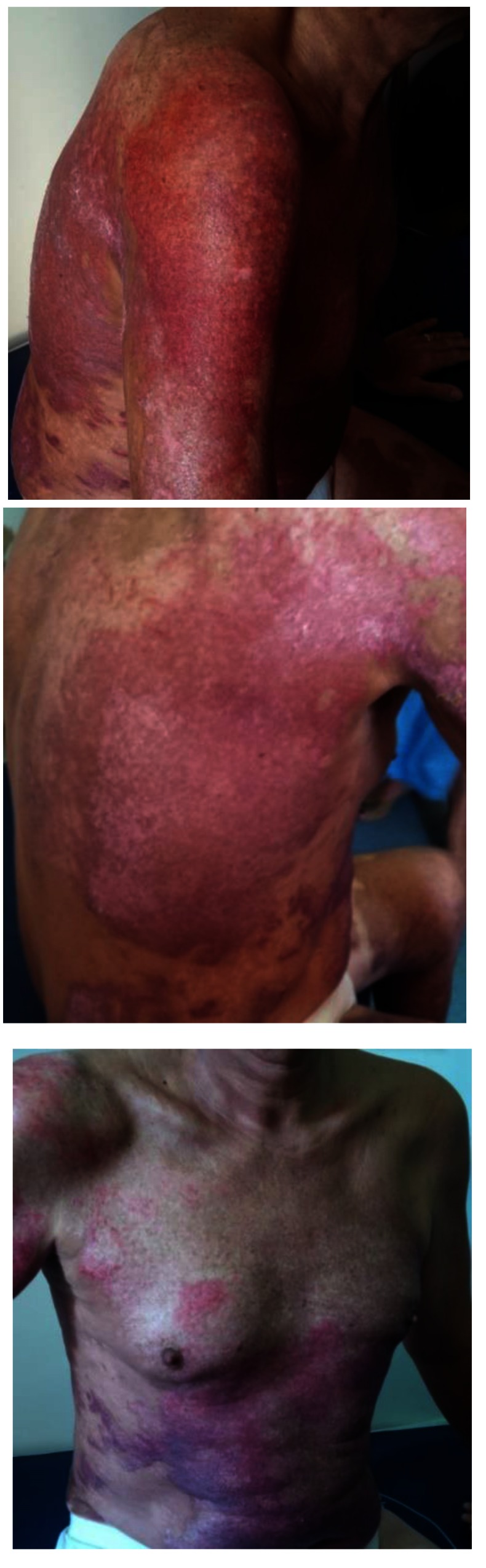
Upper right limb, posterior trunk and respectively anterior trunk of the patient, with healed burns, post-burn “nice-looking”, red, pinkish and violet scars, some white coloured areas, too, showing no signs they were prone to hypertrophic/ keloid development – at discharge from our unit, 24 days after the accident

 The burn injuries on his trunk were slowly healing. Some difficulties were encountered, as he refused to comply with our indication of not lying only on the back, so he developed small eschars on the heels, in spite of the special mattress and of the staff turning him on the sides and on the belly, as indicated in such cases. Once more silver dressings were applied, that time Atrauman Ag. The lesions were not exudative anymore, but still a bit wet and the patient’s body healing reserves were quite depleted, in spite of the administered vitamins and hyper-protein diet, so the epithelization process needed to be boosted. In addition, we thought that a mesh-like dressing, as the one we used, would help. After another week of daily psychological therapy sessions with the psychologist in our unit, he started walking by himself: it was a miracle for us to see him like that. He was discharged completely healed, with still evolving scars that did not show any sign of turning pathological. The small eschars on his heels were slowly healing, too. He was recommended to use scar gel (with onion extract and another one that was silicone based) (**Fig. 7-9**).

**Fig. 10-11:**
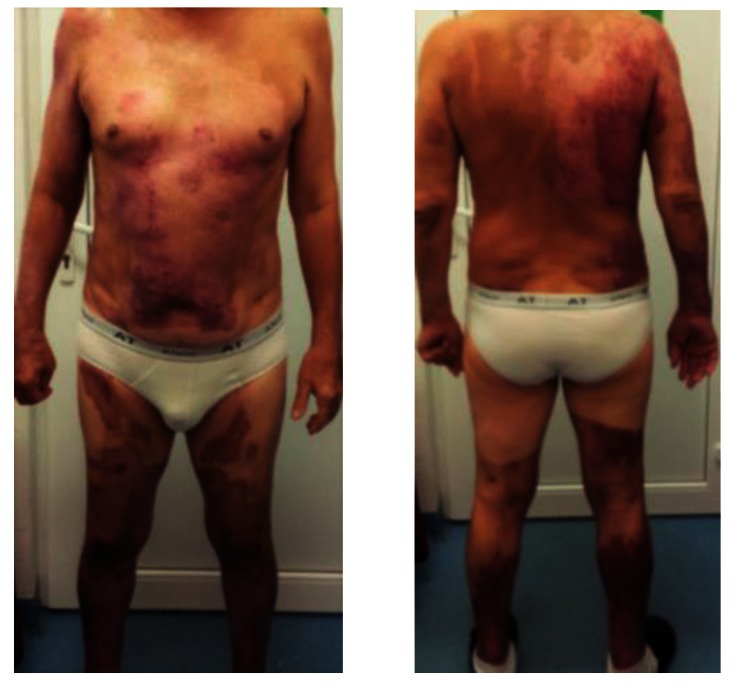
Post-burn scars, after approximatively 3 months from discharge. We noticed that some areas had severe discoloration (were turning either lighter or darker than the surrounding skin). Some areas, like the ones on the right posterior hemi-trunk, right arm and anterior thorax were starting to turn to pathological scars

After 3 months from the discharge, the patient’s scars showed signs of turning hypertrophic/ keloid in some regions: the right scapular area, the right flank, some dot-like areas on the back and on the anterior thorax, also on the right arm (**Fig. 10-11**). 

He started wearing special garments - vest and bandeau on the right arm for pressure therapy. The performance of Corticoid (Kenalog A) injections were also started in the pathological scars. At that point, almost after a year they were slowly turning softer and decreasing their projection. However, we are still performing corticoid injections – it is the sixth monthly session and there are significant signs of improvement as far as the itching and the softness of the scars are concerned, but still relatively minor changes in their size and projection. We plan to pursue the injections until the scars have acceptable appearance and cause minimum discomfort to the patient. He himself declared that he is not very interested in the esthetic appearance, but more in the functional impact of the scars on his lifestyle.

**Case 2**

**Fig. 12:**
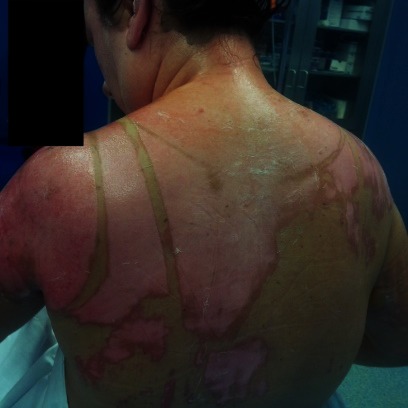
Burn wounds (2nd degree) on the posterior trunk, shoulders and posterior cervical area at the admission in our unit (~ 12 hours after the accident, after surgical debridement in another hospital)

**Fig. 13:**
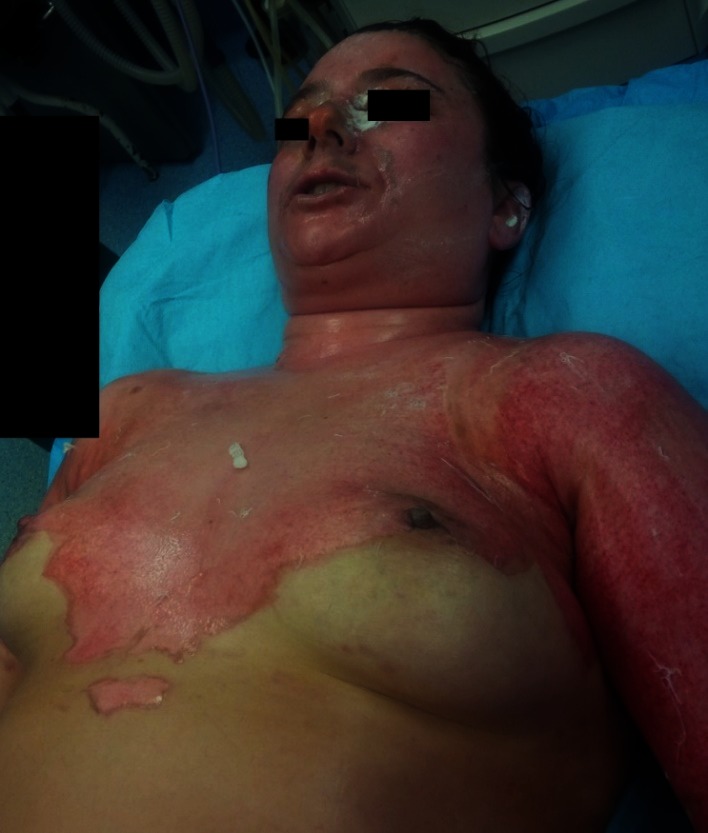
Burn wounds (2nd and 3rd degree) on the face, anterior cervical area, anterior trunk, shoulders, and posterior cervical area at the admission in our unit (~ 12 hours after the accident, after surgical debridement in another hospital)

Patient F.A., female, 35 years old, suffered a household accident – a boiler with hot liquid exploded and splashed her, resulting in II and III degree burns on ~25% TBSA as it follows: the whole face, including the ears, circular burns on the cervical area, and insular ones on the right upper arm, left upper arm, left forearm and upper third of the trunk on both sides (anterior and posterior) (**Fig. 12**,**13**).

The patient was admitted in our unit at about 12 hours from the injury, after first being treated in a smaller hospital, outside Bucharest, where a surgical debridement of the wounds was performed and dressings with Silver sulfadiazine cream (Dermazin) were applied. She displayed burn wounds that did not hurt much, with pink dermis alternating with some whitish areas. We were concerned that the lesions were not painful, but it was probably due to the shock and the medication she had received, as they proved to be more second degree with only some small areas (~ 5% TBSA) of third degree on the left side of the neck and the thorax. She was a very strong, positive, non-smoker, healthy woman, who only had two C-sections and myopia forte in her medical history.

Another cleaning of the burns was first performed and then, on the second day, silver sheets (Acticoat) were applied on the whole burnt area. They were kept in place for a week, the gauze over the foils being changed and being wet at every other day to maintain the product active, as indicated. 

**Fig. 14:**
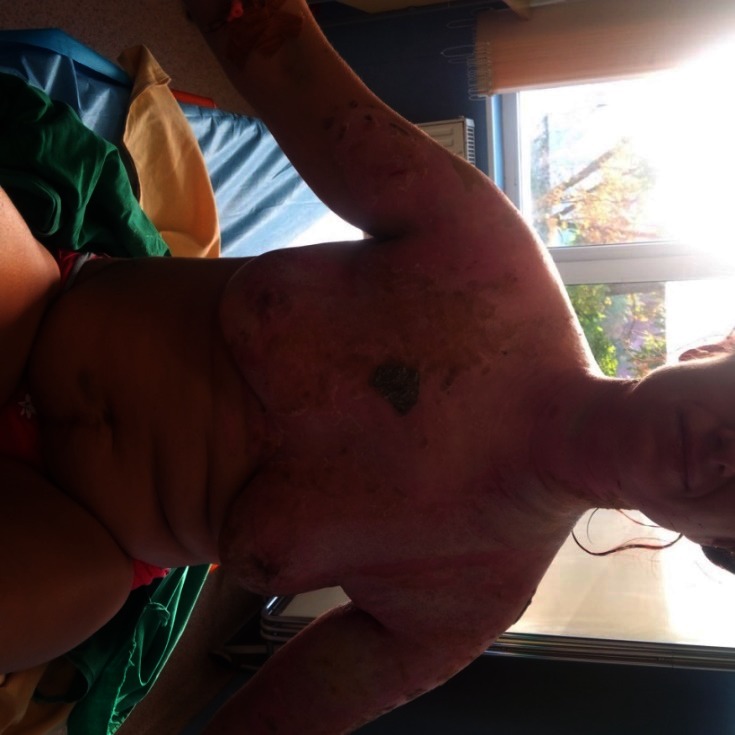
Burns on the anterior thorax and upper limbs healed almost completely after 7 days of having Acticoat sheets applied on them. There were still some small areas like the one on which Acticoat was still adherent (anterior thorax), one on the left breast and the left upper arm, which were still in the process of healing, due to being probably of 2nd B to 3rd degree. The face was completely healed too after 4 days from applying Acticoat

**Fig. 15:**
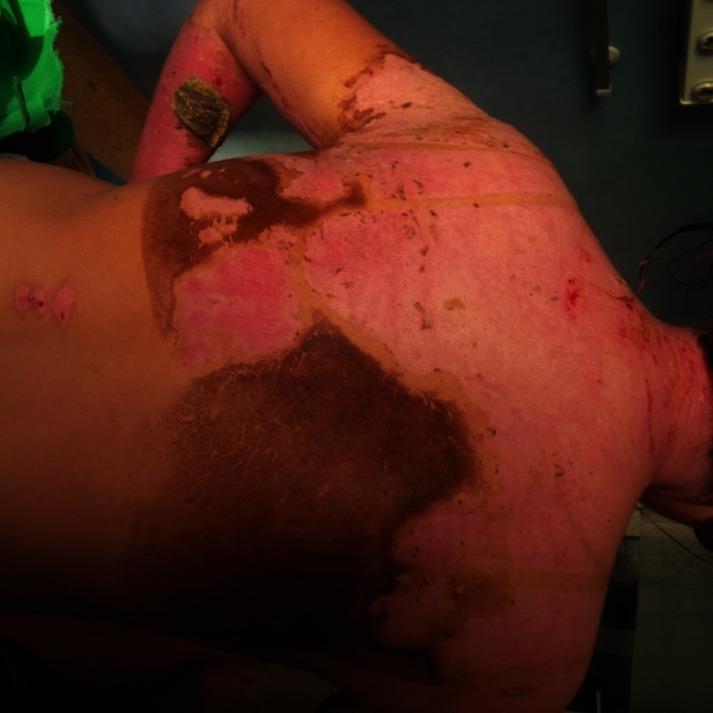
Burns on posterior trunk were completely healed after 5-6 days from applying Acticoat. We noticed that there was a small area left on the volar left arm, where Acticoat could not be removed, as the area was still healing. We also noticed a discoloration of the regular skin, which was normal, due to the contact with the Acticoat sheets that contained silver ions. That darker color usually disappears when washing thoroughly, eventually with a sponge. However, it was completely harmless

The results were the following: on the fifth day, the face and ears were completely healed and the removal of the Acticoat sheets that had already detached themselves from the skin was started. The back side of the trunk and neck were ~ 95% healed with only a couple of small areas that were still healing (**Fig. 14**,**15**). 

After removing all the sheets, both the patient and us were very happy to notice that the upper right arm and the left forearm as well as the anterior part of the neck and most areas on the breasts were healed, so there were just ~ 5%TBSA more to go.

**Fig. 16-18:**
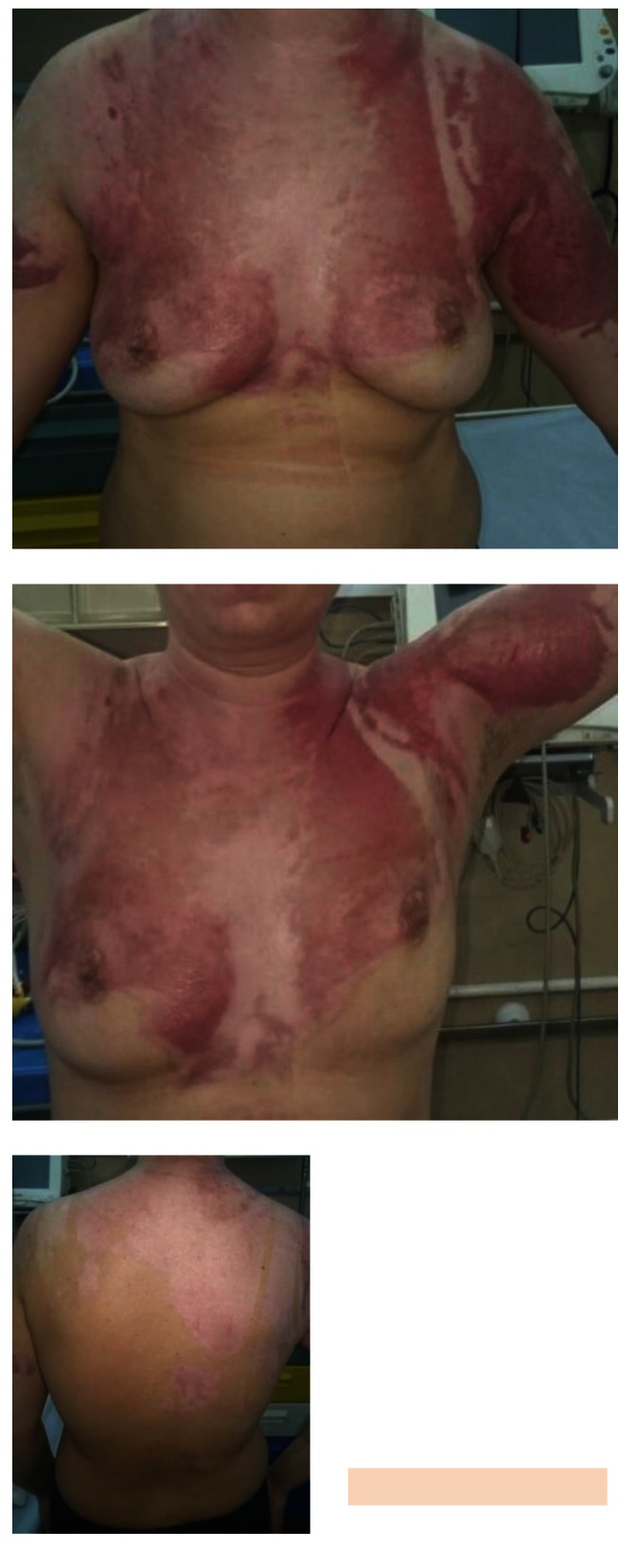
Post-burn pinkish scars on anterior thorax and upper limbs that produced no functional limitation and showed no tendency of turning pathological. White scars on posterior trunk. All of them at ~ 7 months from the accident

The patient came to several check-ups displaying “nice-looking” scars, that did not have any tendency to pathological evolution, in spite of being situated in the keloid prone areas (thorax, shoulders, face and ears) (**Fig. 16-18**).

## Discussion

Some people would ask: 

-Why silver?

“The topical antimicrobial agent silver has been used for hundreds of years in wound care. For example, silver has been used to prevent or manage infection in its solid elemental form (like silver wire placed in wounds), as solutions of silver salts used to cleanse wounds (like silver nitrate solution), and more recently as creams or ointments containing a silver–antibiotic compound (silver sulfadiazine (SSD) cream). Silver nitrate solution is less widely used nowadays, but SSD cream has been an important part of burns management for many years. SSD cream, however, is relatively short-acting, requires reapplication at least daily, and is time-consuming and messy to apply and remove [**2**]”.

-Why a sheet, and not an ointment? 

It has been long known that SSD or silver based ointments (like Flammazine, Dermazine or Flammacerium) have been and are still used in the treatment of burn wounds, silver being their key ingredient, but, there is a difference between the silver sheets and the silver-based topical solutions/ ointments: the topical solutions and ointments create a cream-fibrin adherent layer onto the burn wound, not allowing the plastic surgeon to monitor the evolution of the wound plus the ointments are usually a very good environment for bacteria like pseudomonas, thus promoting in some degree the colonization of the burn, after the silver core becomes inactive. This is why the dressings must be changed very often. 

Therefore, the sheets not only allow a better monitorization of the wound, but they also adhere to the surface and come off usually when everything underneath is healed. The patient also needs less changing of dressings, as the sheets can be held up in place for 3 to 7 days, depending on the type of product and the particularities of the case.

The sheets also have another advantage: being a net-like structure they allow a proper drainage of the exudate produced by the burn lesions, while still keeping the burn moist. Compared to the foils, the creams/ ointments create a layer that does not allow proper ventilation, thus resulting in a sludgy surface on the burn lesion. 

The main disadvantage of the sheets are that not everyone can apply them, one must know how to apply them correctly, how to maintain them active, but mainly how to make the right decision and have the necessary experience to state on which areas their presence would be effective. Another great disadvantage is their cost: silver sheets are expensive so one must be judicious when using them.

Evidence based medicine showed the following facts:

“ActicoatTM (with Nanocrystalline Silver) dressing is an effective antimicrobial barrier dressing. The nanocrystalline coating of silver rapidly kills a broad spectrum of bacteria in as little as 30 minutes. Acticoat dressing consists of three layers: an absorbent inner core sandwiched between the outer layers of the silver coated, low adherent polyethylene net. Nanocrystalline silver protects the wound site from bacterial contamination, while the inner core helps maintain the moist environment optimal for wound healing [**3**]”. 

At this point, Acticoat is one of the best sheet like silver dressings, which when moistened with sterile water, releases silver ions onto the wound surface, destroying within 30 minutes both Gram positive and negative bacteria as well as Vancomycin resistant enterococci and Methicillin resistant S. aureus [**3**]. “If re-moistened, Acticoat produces a controlled release of clusters of silver cations onto the wound, for up to 3 days (if using Acticoat™) or 7 days (if using Acticoat 7). Research has demonstrated that sustained-release silver products have a bactericidal action providing effective management of odor and exudate, thus reducing the risk for colonization, and preventing infection [**4**]”. 

The action is accomplished by the silver ions binding to tissue proteins causing a structural change in the bacterial cell membranes. The silver then binds and denaturizes the bacterial DNA and RNA, thus inhibiting replication [**3**]. Acticoat dressings have been found to be less painful than SSD cream dressings. Acticoat compared with SSD cream reduces burn wound cellulitis. Research revealed that Acticoat has an anti-inflammatory effect through metalloproteinases, allowing optimal epithelialization [**5**]. SSD cream has been found to have pro-inflammatory properties and shown to cause leucopenia [**3**].

-Does silver improve the healing rate? 

“The aim of treatment with silver dressings is to reduce wound bioburden, treat local infection, and prevent systemic spread: their main purpose is not to promote wound healing directly. Clinical guidelines recommend that silver dressings are used for wounds where infection has already established or an excessive wound bioburden delays healing [**2**]”. As we know, all burns are contaminated as they arrive to the emergency unit, the skin barrier being destroyed, even common germs producing infections. Therefore, this is actually the major problem in case of severe and extensive burns, that is why one of the priorities in treating burns is to reduce the bioburden and prevent infection and sepsis, especially in extensive lesions. Thus, silver – as an antibacterial agent - promotes healing in an indirect manner, because an infected wound cannot and will not heal as quickly as a clean one.

-Is it possible to be toxic for the patient?

“Some in vitro studies have found that some silver-containing dressings are cytotoxic to keratinocytes and fibroblasts, and delay epithelialization in animal wound models. Conversely, other studies have found some silver products not to be toxic and have suggested that silver has actions that may promote healing [**2**]”. Some of the ions from Acticoat for instance, color the skin and have antibacterial action, while others have “pro-healing effects” [**6**]. 

“Silver sulfadiazine is absorbed into the skin, where it forms a reservoir of silver ions, which are then released into the tissues [**6**]”. The absorption of silver is influenced by the degree of the burn wound, thus being greater in partial thickness lesions, due to the more abundant vascular supply. It should not be forgotten that leukopenia could appear in patients after silver sulfadiazine was applied, but still the benefits of using silver products are higher than the disadvantages: silver prevents infection, promotes healing, and has anti-inflammatory effects.

-Are there any germs that are resistant to silver ions?

At this point, it appears that there are no silver resistant bacteria and that one cannot develop resistance to it. “For many years, silver sulfadiazine cream has been demonstrated as effective in the treatment of burn wounds and has been the standard of wound care […]. More recently, many silver dressings have been developed for topical use in acute and chronic wound care. While the development of antibiotic resistance is an alarming concern in clinical practice, the advantage of silver is that it has minimal bacterial resistance. Therefore, many dressings with a broad range of components and material characteristics have been silver-coated and are available for therapeutic use. Acticoat™ (Smith & Nephew, Largo, FL) was one of the first silver-coated dressings on the market with a clinical indication for burn wounds. Most treatment experience for pediatric and even neonatal burns has been gained with Acticoat. Subsequently, the silver sulfadiazine impregnated hydrocolloid wound dressing, Urgotul® SSD (LaboratoiresUrgo, Chenove, France), and the silver-coated foam, Contreet Ag® (Coloplast, Minneapolis, MN), became available on the market. Several in-vitro studies confirmed the effectiveness of these silver dressings against a broad range of Gram-negative and Gram-positive bacteria.[…] All silver dressings are comparably effective against a broad range of bacteria. Many studies claim advantages in each tested silver dressing in acute wounds [**7**]”. 

The truth is somewhere in the middle, all silver based products being comparably effective. The problem is that usually silver based dressings are very expensive, being difficult for hospitals in some countries to sustain such expenses, thus leading to the following questions.

Do silver sheets decrease the hospitalization period and costs?

Are they cost effective or not?

An employee in a public hospital tends to be very cost-conscious, as they have to motivate their request for Acticoat in a particular case. “A number of studies have found that silver dressings are associated with factors that may be beneficial in terms of cost effectiveness, as for example:

■ reduced time to wound healing

■shorter hospital stays

■ reduced dressing change frequency

■ reduced need for pain medication during dressing change

■ fewer MRSA bacteraemias resulting from MRSA-infected wounds [**2**]”. 

In various studies performed on burn patients, Acticoat has been shown to shorten in-patient stay compared to patients treated with SSD. The resulting decrease in bedtime and subsequently nursing time in this study means that Acticoat has been demonstrated to lower overall treatment costs compared to silver sulfadiazine in the high cost burns arena [**8**,**9**]. 

“Healing remains the primary goal of partial-thickness burn care, yet […]” most studies “confirm the importance of limiting costs while improving patient comfort and reducing dressing change frequency. Partial-thickness burns dressed with either of 2 silver dressings healed within the 15-day time frame previously reported for silver sulfadiazine gauze-dressed burns [**10**,**11**]”. 

## Conclusion

“Silver based dressing products as well as antimicrobial dressings are recommended for the following types of burns:

- Contaminated burns

- Clinically infected burns

- Deep or full thickness burns

- Burns of mixed or unknown depth

- Minor burns with larger surface areas

In superficial pediatric burn injuries, silver dressing products such as Acticoat is the preferred dressing choice due to the benefits of reduced dressing frequency [**12**]”. 

The purpose of this paper was not to explain the way silver dressings work, this being basic knowledge, nor to advocate for the use of a particular silver-based dressing or ointment, but to merely show our experience with these modern dressings and to pinpoint some interesting topics, which are still investigated worldwide. **Table 1** shows a large variety of silver-based dressings, available nowadays, some of their features being pointed out.

**Table 1:** A large variety of silver-based dressings [**12**]

Dressing	Information and use	Type of wound
Acticoat	A conformable silver antimicrobial barrier dressing with a 3 or 7 day efficacy.	Acute Burns
	A 3-layered dressing consisting of an absorbent rayon/ polyester layer sandwiched between 2 outer layers of silver-coated polyethylene net.	Partial thickness burns
	The silver is in nanocrystalline form, and when moistened is released from the dressing onto the wound.	Infected, contaminated or colonized burns
	Can be left intact for 3 or 7 days depending on the type selected	
	Needs moist secondary dressings to activate the silver	
Acticoat Flex	A knitted, flexible polyester weave, coated with nanocrystalline silver.	Acute Burns
	Stretches too accommodate mobility	Partial thickness burns
	Open weave allows fluid and exudate migration	Infected, contaminated or colonized burns
Acticoat Absorbent	A silver coated highly absorbent alginate dressing with a 3-day antimicrobial efficacy and 7-day wear time.	Recommended for use in the same burn wounds as other Acticoat products but those with a large amount of exudate
	Helps prevent infection, in turn facilitating wound healing	
	Reduces risk of colonization	
	Easy to use and highly conformable	
Mepilex Ag	Silver based silicone which uses Safetac technology	Superficial and mid dermal burns
	Does not adhere to underlying wound bed, minimizing pain associated with dressing changes	Colonized but NOT infected burns
	Conforms well and it is easy to cut to size or shape for wounds in difficult to dress areas.	
Aquacel Ag	Silver-based hydrofibre	Partial thickness burns
	Silver in the dressing kills wound bacteria held in the dressing	Moderate to highly exuding wounds, which are infected or at risk of infection
	Provides an antimicrobial barrier to protect the wound bed	
	Absorbs high amounts of wound fluid and bacteria and creates a soft, cohesive gel that intimately conforms to the wound	
	Colonized but not infected burns	
	Not suitable for over joints	
Silver Sulfadiazine	Topical silver based cream that has a broad antimicrobial activity against both Gram Positive and Gram-negative organisms	Dry wounds
	The cream also contains Chlorhexidine Gluconate that is a disinfectant, which is active against a wide range of vegetative Gram Positive and Gram Negative bacteria	Full thickness burns
	Not suitable for the face	Used on infected or contaminated wounds
Allevyn Ag	Antimicrobial hydrocellular foam dressing with soft gel adhesive	Moderately to highly exuding wounds on patients with fragile or sensitive skin where infection or infections risk needs to be managed.
Biatain Ag	Non-adhesive Foam Dressings with silver	Moderately to highly exudating partial thickness burns
	Prepared with hydro-activated silver, which is released onto the wound during wear	
Antimicrobial Dressings		
Dressing	Information and use	Type of wound
Flaminal	Alginate gel with antimicrobial properties	Flaminal Forte: medium to highly exudating wounds
	Requires suitable secondary dressing and can be left intact for 1–4 days.	Flaminal Hydro: lightly exudating wounds

In our experience, we have only used some of the above-mentioned silver sheets, but we were very happy with the results. The fact that the sheets stay on for a couple of days and the dressings do not need to be changed that often is an advantage both for the physician and the patient, decreasing the needs for sedation and discomfort, thus reducing the amount of used materials, and the subsequent costs. It is also very important that the foils are very adherent and that the patient can move freely, without having an ointment and gauze that keeps becoming sludgy, dripping or getting looser. We only showed some cases, the more interesting ones, but Acticoat was used on more patients. They were all very happy and told us that they had minor discomfort when applying Acticoat, and very little pain afterwards or when changing the dressings, compared to other areas where Flammazine was applied. Overall, it was observed that the bio-burden in these patients was lower and they healed nicely. Thus, there was no need for lower doses of antibiotic treatment, this being one of the main goals in medicine. As germs are becoming more and more dangerous and multi-resistant, we think that not having to use any antibiotic or reducing the doses or the number of days of antibiotic treatment is of great importance. Having an “Ace up the sleeve” like these silver sheets in the fight against infection is essential. 

Many questions have been raised and issues are still coming up when using these types of sheets, but they still have not been answered yet. Numerous ongoing research trials are trying to explain different mechanisms or provide answers.

For instance, it is very interesting to observe the fact that in spite of using the same type of silver sheets in the above-mentioned cases, the outcome as far as scar appearance is concerned, is very different. In spite of being very helpful and effective in the treatment of burns, silver sheets do not prevent pathological scarring. Doing a lot of reading on the subject and looking online, we have not been able to find any studies concerning the impact of silver sheets in the aesthetic outcome of burn wounds, nor concerning the influence of these dressings on scars turning pathological or not. However, it is strange that none of these aspects have been researched or published yet. We believe that this would be an interesting topic for a future research clinical trial and several papers.

**Conflict of interest **

The authors declare they have no conflicts of interest. 

**Disclosures **

The authors declare they have nothing to disclose.
